# Association of Full Breastfeeding Duration with Postpartum Weight Retention in a Cohort of Predominantly Breastfeeding Women

**DOI:** 10.3390/nu11040938

**Published:** 2019-04-25

**Authors:** Muna J. Tahir, Jacob L. Haapala, Laurie P. Foster, Katy M. Duncan, April M. Teague, Elyse O. Kharbanda, Patricia M. McGovern, Kara M. Whitaker, Kathleen M. Rasmussen, David A. Fields, Lisa J. Harnack, David R. Jacobs, Ellen W. Demerath

**Affiliations:** 1 Division of Epidemiology and Community Health, University of Minnesota, Minneapolis, MN 55454, USA; jacob.l.haapala@healthpartners.com (J.L.H.); fost0112@umn.edu (L.P.F.); harna001@umn.edu (L.J.H.); jacob004@umn.edu (D.R.J.J.); ewd@umn.edu (E.W.D.); 2 Department of Pediatrics, University of Oklahoma Health Sciences Center, Oklahoma City, OK 73104, USA; Katy-Duncan@ouhsc.edu (K.M.D.); April-Teague@ouhsc.edu (A.M.T.); David-Fields@ouhsc.edu (D.A.F.); 3 HealthPartners Institute, Minneapolis, MN 55425, USA; elyse.o.kharbanda@healthpartners.com; 4 Division of Environmental Health Sciences, University of Minnesota, Minneapolis, MN 55455, USA; pmcg@umn.edu; 5 Department of Health and Human Physiology, University of Iowa, Iowa City, IA 52242, USA; kara-whitaker@uiowa.edu; 6 Department of Epidemiology, University of Iowa, Iowa City, IA 52242, USA; 7 Division of Nutritional Sciences, Cornell University, Ithaca, NY 14853, USA; kathleen.rasmussen@cornell.edu

**Keywords:** full breastfeeding, postpartum, weight retention, obesity

## Abstract

Full breastfeeding (FBF) is promoted as effective for losing pregnancy weight during the postpartum period. This study evaluated whether longer FBF is associated with lower maternal postpartum weight retention (PPWR) as compared to a shorter FBF duration. The MILK (Mothers and Infants Linked for Healthy Growth) study is an ongoing prospective cohort of 370 mother–infant dyads, all of whom fully breastfed their infants for at least 1 month. Breastfeeding status was subsequently self-reported by mothers at 3 and 6 months postpartum. Maternal PPWR was calculated as maternal weight measured at 1, 3, and 6 months postpartum minus maternal prepregnancy weight. Using linear mixed effects models, by 6 months postpartum, adjusted means ± standard errors for weight retention among mothers who fully breastfed for 1–3 (3.40 ± 1.16 kg), 3–6 (1.41 ± 0.69 kg), and ≥6 months (0.97 ± 0.32 kg) were estimated. Compared to mothers who reported FBF for 1–3 months, those who reported FBF for 3–6 months and ≥6 months both had lower PPWR over the period from 1 to 6 months postpartum (*p* = 0.04 and *p* < 0.01, respectively). However, PPWR from 3 to 6 months was not significantly different among those who reported FBF for 3–6 versus ≥6 months (*p* > 0.05). Interventions to promote FBF past 3 months may increase the likelihood of postpartum return to prepregnancy weight.

## 1. Introduction

Pregnancy is a period of rapid weight gain and change in body composition [1] as maternal metabolism accommodates the demands of a growing fetus [2]. Gestational weight gain within the recommended ranges based on prepregnancy body mass index (BMI) is important for optimal fetal development [1,3] and accretion of fat depots necessary to support the energy cost of lactation [2]. Unfortunately, 47% of women in the United States have excessive gestational weight gain [4] and 13–20% fail to return to their prepregnancy weight after delivery, weighing approximately 5 kg more at 6–18 months postpartum compared to prepregnancy [5,6]. Excessive postpartum weight retention (PPWR) may in turn initiate a vicious cycle of obesity among women during their reproductive years and contribute to future unhealthy pregnancies, diabetes, and cardiovascular disease [1].

Many factors influence variation in PPWR, including maternal prepregnancy BMI, gestational weight gain, parity, age, race/ethnicity, education, diet, physical activity, and breastfeeding duration [7,8,9,10]. Breastfeeding is of particular interest due to its established physical and psychological benefits for both mother and infant during the postpartum period and beyond [11,12,13]. Prolonged breastfeeding may theoretically promote postpartum weight loss owing to the energy expenditure requirements of lactation [14] and mobilization of pregnancy-related accumulated fat stores [2]. 

To date, studies examining the association between breastfeeding and PPWR or postpartum weight loss have yielded inconclusive findings [15,16,17]. In a recent systematic review, Neville et al. concluded that, compared to other forms of infant feeding, there was insufficient evidence indicating that breastfeeding is directly associated with postpartum weight change [18]. Of particular concern is the issue of confounding, whereby women who breastfeed longer are more likely to have intended to breastfeed [19], have greater social support [11], and have lower prepregnancy BMI [20] and gestational weight gain [21] than women who breastfeed for short durations, never breastfeed or breastfeed only partially. All these factors are directly associated with PPWR [7,8,9,10]. Most studies included in this comprehensive review had relatively few women who were exclusively breastfeeding to 6 months as recommended, and/or did not adjust for important potentially confounding variables. They also often relied on self-reported maternal weight and lacked repeated measurements to assess patterns of weight retention over time, both of which decrease precision. Additional studies that can characterize the relationship between longer versus shorter full breastfeeding (FBF) and PPWR, including in women who are strongly committed to and initiate full breastfeeding, are therefore warranted.

In this exploratory study, we aimed to examine the association between FBF duration and maternal PPWR from 1 to 6 months postpartum using repeated measures of FBF and objectively measured maternal anthropometry within a cohort of healthy, predominantly breastfeeding women. We hypothesized that, compared to mothers who fully breastfed for 1–3 months and 3–6 months, those who fully breastfed for ≥6 months would exhibit lower PPWR from 1 to 6 months, and from 3 to 6 months, respectively.

## 2. Materials and Methods 

### 2.1. Study Population

The Mothers and Infants Linked for Healthy Growth (MILK) study is an ongoing prospective cohort of mother–infant dyads recruited from Minneapolis, MN and Oklahoma City, OK [22]. Women were included in the study if they: were 21–45 years of age at delivery; had a prepregnancy BMI of 18.5–40.0 kg/m^2^; had a healthy singleton pregnancy (i.e., spent <3 days in the hospital post-delivery for vaginal deliveries and <5 days for caesarean section deliveries); delivered an infant at-term with a birthweight of ≥2500 g but ≤4500 g; and reported an intention to breastfeed exclusively for at least 3 months. Women were excluded from the study if they consumed tobacco or >1 alcoholic drink per week during pregnancy/lactation, had a history of Type 1 or Type II diabetes or current diagnosis of gestational diabetes, were unable to speak or understand English, or if the infant had a known congenital illness affecting feeding and/or growth.

Of the 370 mother–infant dyads enrolled into the MILK study (all fully breastfeeding at 1 month postpartum), 32 were excluded due to missing information on breastfeeding status at 3 or 6 months or maternal prepregnancy weight. The final analytic sample size included 338 mother–infant dyads with complete information on FBF duration, maternal prepregnancy weight, and maternal weight on at least one of the follow-up time points (i.e., at 1, 3 or 6 months postpartum). 

Written informed consent was obtained at baseline, and participants were compensated for completion of each visit. All study protocols were approved by the institutional review boards at the University of Minnesota, HealthPartners Institute, and the University of Oklahoma Health Sciences Center. 

### 2.2. Breastfeeding Status

Maternal breastfeeding status was ascertained at the 1-, 3-, and 6-month study visits. Mothers were asked to report their breastfeeding habits, which were further classified as FBF, mixed-feeding or fully formula feeding. Given our inability to discern “exclusive” (no other solid foods/liquids) from “almost exclusive” breastfeeding (water, vitamins and minerals occasionally provided), we defined FBF as maintaining breastfeeding with <24 oz of formula during the entire time period, and only breast milk for the 2 weeks prior to the visit. Mixed feeding was defined as providing infants with >24 oz of formula for each time period but also some breast milk. Fully formula feeding was defined as providing infants only formula for each time period. We categorized the duration of FBF in terms of the number of completed months of FBF as follows: 1–3 months, 3–6 months, and ≥6 months. The frequencies of various barriers to lactation were queried at the 1-month study visit.

### 2.3. Maternal Anthropometry

Maternal prepregnancy weight (kg) was measured within 6 weeks of conception and abstracted from electronic medical records. Maternal weight was then measured at the 1-, 3-, and 6-month study visits using calibrated digital scales. Standard procedures were followed after cross-training of staff [23]. PPWR was calculated and defined as maternal weight at the 1-, 3-, and 6-month visits minus maternal prepregnancy weight. 

### 2.4. Covariates

Maternal educational attainment (high school/GED/Associates degree, Bachelor’s degree, graduate degree), race/ethnicity (white, other), household income (<$60,000, $60,000–$90,000, >$90,000), physical activity at 3 months postpartum (meets/does not meet moderate-to-vigorous physical activity (MVPA) guidelines of 150 min/week) [24], and frequency of feeds at 1, 3, and 6 months postpartum (≤6, >6 times/day) were self-reported by mothers. Dietary intake was self-reported by mothers at 1 month postpartum using a modified version of the Diet History Questionnaire II to reflect intake during the past month [25]. Maternal age (years), parity (0, 1, ≥2), delivery mode (vaginal, caesarean section), infant birthweight (grams), and infant sex (male, female) were abstracted from the mother’s electronic medical records. Maternal gestational weight gain (kg) was calculated by subtracting maternal prepregnancy weight from weight at delivery (measured within 2 weeks of birth and abstracted from medical records).

### 2.5. Statistical Analyses

Characteristics of the mother–infant dyads were described using raw means ± standard deviations (SD) and raw frequencies stratified by FBF duration. Chi-square tests and one-way ANOVAs were used to compare participant characteristics by FBF duration for categorical and continuous variables, respectively. The purpose of these statistical tests was to identify potential confounding variables. One inclusion criterion for the study included an intention to fully breastfeed for at least 3 months and the presence of breastfeeding support; however, *n* = 23 mothers stopped breastfeeding before 3 months. As such, we examined differences in frequencies of barriers to lactation between women who fully breastfed to 1–3 months and those who fully breastfed for ≥3 months using chi-square tests.

Linear mixed effects models (PROC MIXED) were then used to test the association of FBF duration with repeated within-subject measures of maternal PPWR at 1, 3, and 6 months postpartum using an unstructured covariance matrix. These models can accommodate unbalanced intervals of measurement, time-dependent and independent exposures/covariates, and provide greater statistical power with serial measurements [26]. We first examined the crude association of FBF duration with maternal PPWR. The model was then adjusted for maternal education, race/ethnicity, household income, MVPA at 3 months postpartum, frequency of feeds (time-varying), age, parity, delivery mode, gestational weight gain, and infant birthweight and sex. The main exposure (FBF duration) was included as a main effect and as an interaction with time (time-varying). All covariates were included as main effects, but we only retained an interaction with time if the corresponding estimate for the interaction had a *p*-value < 0.05. Since maternal prepregnancy weight was part of the outcome variable, it was not included as a potential confounder in adjusted analyses. However, in sensitivity analyses, we adjusted for maternal prepregnancy BMI in final models to assess whether any observed associations persisted after accounting for prepregnancy weight status. In another sensitivity analysis, we included maternal Healthy Eating Index-2015 (HEI-2015) total scores and energy intakes at 1 month postpartum as covariates in the final models. Dietary intake was not included in the main analysis owing to the relatively large number of missing data (approximately 13%). Lastly, we tested whether similar findings were observed in main analyses when maternal postpartum weight loss (defined as maternal weight at 1, 3, and 6 months minus maternal weight at delivery) was utilized as an outcome, rather than PPWR.

Because longitudinal mixed effects regression model estimates can be difficult to interpret in the presence of time interactions, we derived and plotted the adjusted means ± SEs for PPWR from the linear mixed model analysis specified above for each FBF group and each time point. Next, a difference-of-differences analysis was conducted, such that the change in adjusted means of weight retention over time (slopes) within each FBF duration category (e.g., PPWR at 1 month minus PPWR at 6 months) were compared across FBF duration categories. Specifically, differences in PPWR from 1 to 6 months were compared for mothers who fully breastfed for 3–6 and ≥6 versus 1–3 months and differences in PPWR from 3 to 6 months were compared for mothers who fully breastfed for 3–6 versus ≥6 months, with corresponding *p*-values derived from *t*-tests presented. Statistical analyses were conducted using SAS, version 9.4 (SAS Institute, Inc., Cary, NC, USA).

## 3. Results

### 3.1. Participant Characteristics

Mothers included in the analyses were predominantly white, highly educated, and multiparous; most mothers delivered vaginally, met MVPA guidelines at 3 months postpartum, and fully breastfed for ≥6 months (Table 1). By the 6-month visit, less than half of the women who stopped FBF at 1–3 months were partially breastfeeding (48% were mixed feeding), while 73% of the women who continued to fully breastfeed for 3–6 months were still partially breastfeeding (mixed feeding). Maternal age, race, weight retention at 6 months postpartum, frequency of feeds at 3 and 6 months postpartum, and infant birthweight differed significantly by FBF duration, such that mothers who fully breastfed for longer durations were more likely to be older, white, retain less weight, feed more frequently at 3 and 6 months, and give birth to heavier infants (*p* < 0.05). There were no significant differences in delayed lactogenesis, milk flow, milk supply, sore, cracked or bleeding nipples, breast engorgement, breast yeast infection, clogged milk ducts, infected or abscessed breasts or breast milk leakage between mothers who fully breastfed for 1–3 months versus ≥3 months (all *p* > 0.20; Table S1).

### 3.2. Full Breastfeeding Duration and Postpartum Weight Retention 

**Figure 1:**
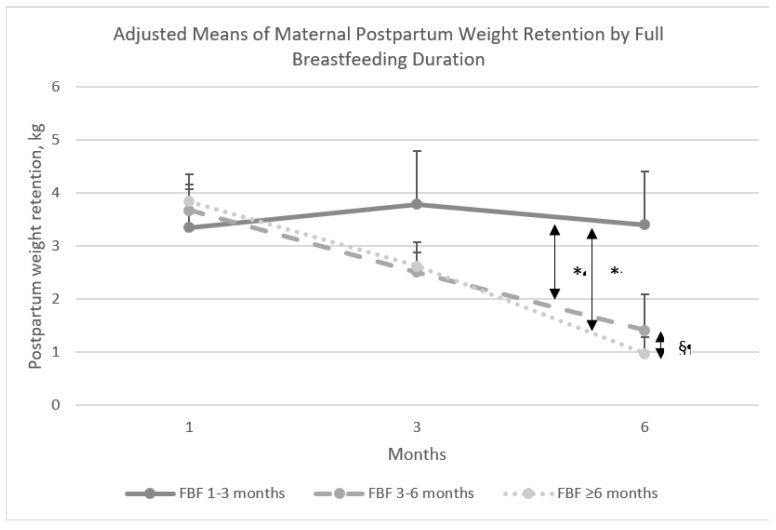
Adjusted means of maternal postpartum weight retention by full breastfeeding duration. Abbreviations: FBF = full breastfeeding; Adjusted for maternal education, race/ethnicity, household income, age, parity, delivery mode, prepregnancy weight, gestational weight gain, frequency of feeds at 1, 3, and 6 months (time-varying), physical activity level at 3 months postpartum, and infant birthweight and sex. * *p* < 0.05 for differences in slopes. § *p* > 0.05 for differences in slopes.

A significant interaction between FBF duration and time was observed, suggesting between-group differences in PPWR over time (Table 2). To illustrate the interaction effect of FBF and time on PPWR, Figure 1 depicts differences in adjusted means ± SE of maternal PPWR from 1 to 6 months postpartum by FBF duration. By 6 months postpartum, mothers who fully breastfed for 1–3, 3–6, and ≥6 months had adjusted means ± SEs for PPWR of 3.40 ± 1.16 kg, 1.41 ± 0.69 kg, and 0.97 ± 0.32 kg. Compared to mothers who fully breastfed for 1–3 months only, those who fully breastfed for 3–6 and ≥6 months retained significantly less weight from 1 to 6 months postpartum (*p* = 0.04 and *p* < 0.01, respectively). Although mothers who fully breastfed for 6 months or longer weighed approximately 0.45 kg less by 6 months postpartum than those who fully breastfed for 3–6 months only, this difference in PPWR at 6 months and slope of change in PPWR from 3 to 6 months was not significant between these two groups (*p* = 0.15). These findings were independent of the statistical effects of numerous potential confounders included in the model. Significant covariate effects were observed for MVPA and maternal age, and time-dependent effects were observed for GWG, maternal education, parity, and mode of delivery (Table S2).

In sensitivity analyses, after including maternal prepregnancy BMI as a potential covariate in the final model examining the association between FBF duration and maternal PPWR, comparable findings were observed for adjusted means and differences in slopes of PPWR over time (data not shown). After additional adjustment for maternal total HEI-2015 scores and energy intake at 1 month postpartum, adjusted means were consistent with those found in the main analyses. In this sensitivity analysis, differences in slopes of change in weight retention from 1 to 6 months for women who fully breastfed to ≥6 versus 1–3 months were similar to those reported above for the main analysis. However, slopes of change in weight retention were no longer significantly greater among those who fully breastfed to 3–6 versus 1–3 months (*p* < 0.10) (data not shown). Lastly, after examining postpartum weight loss (versus PPWR) as an outcome, similar adjusted means and between-group differences in weight loss over time were observed (data not shown). 

## 4. Discussion

In this ongoing prospective cohort of mother–infant dyads, we examined the association of FBF duration with maternal weight retention from 1 to 6 months postpartum. We found that, independent of demographic, reproductive, and lifestyle factors, mothers who fully breastfed for longer durations (i.e., greater than 3 months) had significantly greater reductions in PPWR from 1 to 6 months compared to those who fully breastfed to 1–3 months only. Mothers who fully breastfed to 6 months or longer retained approximately 0.45 kg less weight by 6 months postpartum than those who fully breastfed to 3 months only, but differences between these two groups were not statistically significant. The findings from this exploratory analysis suggest that, among healthy women strongly committed and supported to fully breastfeed, encouraging FBF beyond 3 months could reduce PPWR.

The American Academy of Pediatrics recommends exclusive breastfeeding to 6 months, followed by complementary feeding with continued breastfeeding until 1 year and beyond as mutually desired by mothers and their infants [27]. This practice promotes infant health but also has immediate (postpartum) and long-term health benefits for mothers [11,12,13]. Breastfeeding is hypothesized to mobilize pregnancy-related buildup of visceral and femoral fat stores as a source of energy for milk production [2]. With the concurrent rise in prolactin, lipogenesis is inhibited in peripheral adipose tissue and increases in the mammary glands [28]. Lipoprotein lipase activity in adipocytes (particularly in the femoral region) also surges, resulting in greater weight loss among breastfeeding mothers [29]. Research has demonstrated that maternal total energy expenditure increases by approximately 15–25% during lactation as the production of milk requires an additional 500 kilocalories per day on average [30]. If this excess energy expenditure is not offset by increases in energy intake and decreases in physical activity, the expectation is that mothers will lose weight.

Nonetheless, the 2018 CDC breastfeeding report card suggests that approximately 47% of women in the United States exclusively breastfeed up to 3 months, and only 25% continue to do so up to 6 months with variation between states [31]. A host of social and biological determinants contribute to early cessation of exclusive breastfeeding, including poor family and social support, embarrassment, employment, lactation problems, and perceived lack of sufficient milk supply [32]. Most mothers in the United States do not meet their breastfeeding goals [33], and it is unclear why 23 of the mothers in our highly motivated and supported cohort ceased FBF between 1 and 3 months despite intending to fully breastfeed for at least 3 months. There were no significant differences in early barriers to lactation observed between those with an FBF duration of 1–3 months versus ≥3 months. It is possible that mothers who stopped fully breastfeeding had to return to work, had fewer workplace accommodations for breastfeeding, did not want to pump milk after returning to work or had infants who lost interest in nursing or did not gain enough weight [33]. However, since none of these possible differences are likely to drive postpartum weight loss, they are unlikely to explain the differences we have reported.

Our results on the association between FBF duration and PPWR are in line with those of other studies [15,16,34]. In a cohort of 405 Brazilian women, mean weight retention was approximately 3.6 kg by 9 months postpartum, and longer breastfeeding duration was associated with lower PPWR from 0.5 to 9 months of follow-up [15]. Similarly, Janney et al. reported a mean weight retention of 3.9 kg at 6 months postpartum and lower weight retention from 0.5–18 months postpartum among 110 mothers in the United States who had a longer duration of full and partial breastfeeding [16]. Contrary to our findings, Mullaney et al. reported no association between exclusive breastfeeding and maternal weight change by 4 months postpartum among 470 Irish women enrolled in a prospective cohort study (mean retention at 4 months postpartum was approximately 1.7 kg) [17]. Few studies have had the opportunity to assess the association between prolonged FBF (i.e., up to 6 months) and PPWR due to the limited number of US women who continue to breastfeed beyond 3 months. In one study of over 25,000 mothers in the Danish National Birth Cohort, Baker et al. found that those who breastfed as recommended (i.e., exclusively to 6 months and to any degree for 12 months) had a greater reduction in PPWR at 6 months (regardless of prepregnancy BMI) and at 18 months (among women within the BMI range of 18.5–34.9 kg/m^2^) [34]. 

Discrepancies in findings pertaining to the associations of FBF with PPWR are evident between studies [8,17,35]. Complications in comparisons across research may be attributable to differences in study design, sample sizes (and, accordingly, statistical power), definition and assessment of breastfeeding duration and intensity, time points at which outcomes were assessed and/or periods of collection that were collapsed into one variable, specific outcomes considered and methods used to measure them, quality of exposure and outcome data, duration of follow-up, and confounders included in analyses. It is important to note that our study population is relatively healthy, with 74% of women fully breastfeeding to 6 months (well above the national rates for exclusive breastfeeding) [28] and 55% meeting MVPA guidelines at 3 months postpartum. Among the 63 mothers who breastfed to at least 3 months but not 6 months or more, 73% continued to practice mixed feeding, that is, feeding their infants both breast milk and formula. If only some breastfeeding is required to achieve continued postpartum weight loss, this may explain the similarity in the rate of weight loss from 3 to 6 months in those who breastfed to ≥6 months as compared to only 3–6 months. Further, the mothers in our study are highly educated and had a lower prepregnancy BMI compared to pregnant women in the United States. Our findings of relatively low weight retention by 6 months postpartum even among those with 1–3 months of FBF (3.9 kg) and a nonsignificant difference in PPWR over time between mothers who fully breastfed for 3–6 months versus to 6 months or longer may be attributable to these characteristics. 

Our findings can be interpreted within the context of the strengths and limitations of the study. A strength of our study is that the data were drawn from a contemporary, multisite, prospective cohort with serial measurements from 1 to 6 months postpartum, which provides greater statistical power and allows for descriptions of patterns of weight retention over time. Furthermore, we objectively measured weight at 1, 3, and 6 months postpartum, extracted prepregnancy weight from electronic medical records and used rigorous definitions of FBF in real time, which minimizes recall bias. Our study population is well characterized; thus, we were able to adjust for a range of clinical, sociodemographic, and lifestyle potential confounders obtained from detailed questionnaires or medical records, including key elements of energy balance, such as physical activity and dietary intake. A characteristic that distinguishes this cohort from others is that the primary aim of the study was to assess the lactational programming hypothesis. As such, we focused on maternal obesity and breastfeeding as main exposures and were able to adjust for gestational weight gain and frequency of feeds within a day in our primary analyses.

Our study also has important limitations. The MILK study was designed to address the relationship of maternal weight status to breast milk composition in fully breastfeeding women. We were therefore not able to assess the broader questions of whether PPWR differs among women who ever versus never breastfeed, or between women who partially breastfeed for different durations versus those who fully breastfeed. In addition, most of the women were fully breastfeeding for ≥6 months and the samples of women with shorter FBF durations were relatively small. As such, the precision of estimates for the 1–3 and 3–6 months FBF groups were lower than for the ≥6 months FBF group, and we had reduced statistical power to detect PPWR differences between the three groups. Replication of these findings is necessary in larger cohorts of women who initiate FBF but then cease FBF early to characterize the benefit of FBF in reducing PPWR among women who follow breastfeeding guidelines. The mother–infant dyads included in the cohort were primarily non-Hispanic white. Therefore, the generalizability of our results is limited for other race/ethnic groups [35]. We did not collect maternal anthropometry beyond 6 months and consequently could not examine whether the lower weight retention associated with FBF was maintained in the long-term [36]. We also did not have a sufficient sample size to stratify our analysis by maternal obesity [34]. This drawback is important, given the observed differences in initiation and maintenance of FBF [37] and in fat distribution during pregnancy among normal-weight, overweight, and obese women [38]. Maternal prepregnancy weight was measured and abstracted from medical records within 6 weeks of conception, which may lead to variation in assessment of true prepregnancy weight and introduce measurement error. Although we adjusted for numerous theoretical and empirical confounders in our analyses, there may still be residual confounding or confounding by unmeasured variables. Lastly, we only had measures of important energy balance variables (i.e., diet and physical activity) at select time points. These behaviors tend to correlate strongly over time, but this limitation may have resulted in residual confounding that could have biased the estimates somewhat.

## 5. Conclusions

The perinatal period is one of significant weight and body composition change for women [1]. Consequently, this period is important for preventing excessive gestational weight gain and PPWR, both of which may contribute to future metabolic disease risk. Findings from the present study correspond to some prior research showing that longer FBF duration may be associated with reduced maternal PPWR up to 6 months postpartum [15,16,34]. Coupled with data from other studies, it is evident that, among healthy, predominantly breastfeeding mothers, FBF for prolonged periods (at least 3 months) is associated with significantly less pregnancy weight retention during the postpartum period. FBF for at least 3 months is a potentially modifiable infant feeding choice that not only promotes infant health but also may help to prevent mothers from future development of obesity and cardiometabolic disorders. Studies that identify how to reduce obstacles to prolonged full breastfeeding are critical to advancing the health of both women and their children.

## Figures and Tables

**Table 1:** Demographic, reproductive, and lifestyle characteristics of mother–infant dyads by full breastfeeding duration (*n* = 338).

Participant Characteristics		Full Breastfeeding Duration			
	Total	1–3 Months (*n* = 23)	3–6 Months (*n* = 63)	≥6-Months (*n* = 252)	
	*n* (%) or mean ± SD				*p*-Value
**Maternal Age, years**	30.8 ± 4.1	28.7 ± 4.9	30.7 ± 4.1	31.0 ± 4.0	0.03 *
**Maternal Race**					
White	288 (87)	14 (64)	51 (82)	223 (90)	
Other	44 (13)	8 (36)	11 (18)	25 (10)	<0.01 *
**Maternal Education**					
High school /GED/Associates degree	74 (23)	10 (44)	11 (19)	53 (22)	
Bachelor’s degree	135 (41)	9 (39)	27 (47)	99 (40)	
Graduate degree	117 (36)	4 (17)	19 (33)	94 (38)	0.09
**Household income**					
<$60,000	99 (30)	11 (48)	22 (39)	66 (27)	
$60,000**–**90,000	80 (25)	4 (17)	15 (26)	61 (25)	
>90,000	147 (45)	8 (35)	20 (35)	119 (48)	0.11
**Parity**					
0	141 (42)	13 (59)	21 (35)	107 (43)	
1	128 (39)	4 (18)	29 (48)	95 (38)	
≥2	63 (19)	5 (23)	10 (17)	48 (19)	0.17
**Delivery Mode**					
Vaginal	264 (80)	17 (77)	50 (81)	197 (79)	0.94
Caesarean section	68 (20)	5 (23)	12 (19)	51 (21)	
**Prepregnancy BMI, kg/m^2^**	26.5 ± 5.6	28.1 ± 5.3	26.9 ± 6.9	26.2 ± 5.2	0.24
**Gestational weight gain (kg)**	12.3 ± 6.6	12.2 ± 8.8	11.7 ± 7.0	12.5 ± 6.3	0.69
**Postpartum weight retention (kg)**					
1-month	3.9 ± 5.4	4.2 ± 7.2	3.2 ± 6.5	4.0 ± 5.0	0.56
3-months	2.8 ± 5.5	4.6 ± 7.9	1.9 ± 6.4	2.8 ± 4.9	0.13
6-months	1.4 ± 5.9	4.3 ± 7.8	0.9 ± 6.4	1.2 ± 5.5	0.05 *
**HEI-2015 score at 1-month postpartum**	65.6 ± 8.9	64.8 ± 9.6	63.7 ± 6.3	66.4 ± 8.9	0.16
**Energy intake at 1-month postpartum (kcal)**	1952 ± 761	1666 ± 770	1889 ± 633	1975 ± 666	0.14
**Meets MVPA guidelines at 3-months postpartum (yes)**	178 (54)	13 (62)	25 (40)	140 (57)	0.06
**Frequency of feeds (per day)**					
1-month	9.7 ± 2.1	9.4 ± 2.3	9.4 ± 2.0	9.8 ± 2.1	0.44
3-months	7.6 ± 2.1	4.6 ± 2.1	7.8 ± 2.0	7.7 ± 1.9	<0.01 *
6-months	6.8 ± 2.1	3.6 ± 1.8	6.0 ± 2.8	7.1 ± 1.7	<0.01 *
**Breastfeeding status at 6 months postpartum**					
Fully breastfeeding	252 (74)			252 (100)	<0.01 *
Mixed-feeding	57 (17)	11 (48)	46 (73)		
Fully formula feeding	29 (9)	12 (52)	17 (27)		
**Infant birthweight, (g)**	3534 ± 445	3329 ± 482	3620 ± 425	3530 ± 442	0.03 *
**Infant sex**					
Male	172 (51)	10 (43)	38 (60)	124 (49)	0.22
Female	166 (49)	13 (57)	25 (40)	128 (51)	

Abbreviations: BMI = body mass index; HEI-2015 = Healthy Eating Index-2015; meeting MVPA guidelines: Moderate to vigorous physical activity >150 min/week. * *p* < 0.05 for tests of differences in participant characteristics by full breastfeeding duration using chi-square or one-way ANOVA for categorical and continuous variables, respectively.

**Table 2:** Association of full breastfeeding duration with maternal postpartum weight retention measured from 1 to 6 months postpartum.

	Crude Model (*n* = 338)			Covariate-Adjusted Model ^a^ (*n* = 301)		
	β	95% CI	*p*-Value	β	95% CI	*p*-Value
**FBF duration**						
1–3 months	Ref	Ref		Ref	Ref	
3–6 months	−1.07	−3.67, 1.54		0.33	−1.55, 2.20	
>6 months	−0.24	−2.57, 2.08	0.18	0.49	−1.18, 2.16	0.53
**Time ^c^**						
1–3 months	Ref.	Ref		Ref	Ref	
3 months	0.39	−0.68, 1.46		0.26	−1.34, 1.86	
6 months	0.04	−1.79, 1.86	<0.01	1.97	−0.66, 4.61	0.20
**FBF duration x time ^b,c^**						
FBF 3–6 months at 3 months	−1.60	−2.86, −0.35		−1.60	−2.95, −0.25	
FBF 3–6 months at 6 months	−2.27	−4.41, −0.13		−2.32	−4.54, −0.09	
FBF ≥6 months at 3 months	−1.55	−2.67, −0.43	0.03	−1.66	−2.86, −0.45	0.04
FBF ≥6 months at 6 months	−2.76	−4.67, −0.85		−2.92	−4.90, −0.94	

Abbreviations: CI = confidence interval; FBF = full breastfeeding. ^a^ Adjusted for maternal education, race/ethnicity, household income, age, parity, delivery mode, prepregnancy weight, gestational weight gain, frequency of feeds at 1, 3 and 6 months (time-varying), physical activity level at 3 months postpartum, and infant birthweight and sex. ^b^ Reference category = FBF 1 month. ^c^ Reference category = time 1 month.

## Supplementary Materials

The following are available online at https://www.mdpi.com/2072-6643/11/4/938/s1, Table S1. Differences in barriers to lactation among mothers who fully breastfed to one month only and those who fully breastfed for more than one month (*n* = 338); Table S2: Association of maternal and infant covariates with maternal postpartum weight retention from 1 to 6 months postpartum (*n* = 301).
