# Supplementary material for: Association of Full Breastfeeding Duration with Postpartum Weight Retention in a Cohort of Predominantly Breastfeeding Women
Source: Nutrients. 2019 Apr 25;11(4):938. doi: 10.3390/nu11040938 (PMC6520964; doi:10.3390/nu11040938)
Supplement: Supplementary file 1 [file nutrients-11-00938-s001.pdf]

nutrients

MDPI

Table S1. Differences in barriers to lactation among mothers who fully breastfed to one month only and those who fully breastfed for more than one month (n = 338).

Maternal Breastfeeding Concern	Full breastfeeding duration		p-Value
	**1 month (n = 23)**	**>1 month (n = 315)**	
	***N* (%)**		
**Delayed lactogenesis**			
No	22 (96)	299 (95)	0.88
Yes	1 (4)	16 (5)	
**Trouble with milk flow**			
No	21 (91)	304 (97)	0.21
Yes	2 (9)	11 (3)	
**Insufficient milk supply**			
No	21 (91)	301 (96)	0.35
Yes	2 (9)	14 (4)	
**Sore, cracked or bleeding nipples**			
No	10 (43)	139 (44)	0.95
Yes	13 (57)	176 (56)	
**Engorged breasts**			
No	15 (65)	171 (54)	0.31
Yes	8 (35)	144 (46)	
**Breast yeast infection**			
No	23 (100)	312 (99)	0.64
Yes	0	3 (1)	
**Clogged milk ducts**			
No	22 (96)	293 (93)	0.63
Yes	1 (4)	22 (7)	
**Infected or abscessed breasts**			
No	22 (96)	310 (98)	0.33
Yes	1 (4)	5 (2)	
**Breast milk leakage**			
No	21 (91)	288 (91)	0.98
Yes	2 (9)	27 (9)	

Table S2. Association of maternal and infant covariates with maternal postpartum weight retention from 1 to 6 months postpartum (n = 301).

	Type 3 Tests of Fixed Effects			
**Variable**	**Numerator Degrees of Freedom**	**Denominator Degrees of Freedom**	**F Value**	***p*-Value**
**FBF duration**	2	285	0.64	0.53
**Time**	2	285	1.63	0.20
**FBF duration *Time**	4	285	2.58	0.04 *
**GWG**	1	285	299.55	<0.01 *
**GWG*Time**	2	285	13.72	<0.01 *
**Maternal Education**	2	285	1.11	0.33
**Maternal Education*Time**	4	285	4.55	<0.01 *
**Maternal Race**	1	285	0.14	0.71
**Parity**	2	285	1.41	0.25
**Parity*Time**	4	285	3.18	0.01 *
**Frequency of Feeds**	1	285	2.30	0.13
**Physical Activity at 3 Months Postpartum**	1	285	7.16	0.01 *
**Delivery Mode**	1	285	0.01	0.91
**Delivery Mode*Time**	2	285	3.07	0.05 *
**Household Income**	2	285	0.68	0.51
**Maternal Age**	1	285	4.15	0.04 *
**Infant Sex**	1	285	0.00	0.96
**Infant Birthweight**	1	285	3.08	0.08

Abbreviations: FBF=full breastfeeding; GWG=gestational weight gain. * $p < 0.05$.
